# Supplementary material for: Of power and despair in cetacean conservation: estimation and detection of trend in abundance with noisy and short time-series
Source: PeerJ. 2020 Aug 7;8:e9436. doi: 10.7717/peerj.9436 (PMC7416721; doi:10.7717/peerj.9436)
Supplement: Supplemental Information 1 [file peerj-08-9436-s001.zip › 20200514_PeerJ_AuthierEtAl_suppl/AppendixI.pdf]

Supplementary Material to "Of Power and Despair in Cetacean Conservation: Estimation and Detection of Trend in Abundance with Noisy and Short Time-Series" by Authier et al.

May 14, 2020

This supplementary material provides further details on the Monte Carlo analyses carried by Authier *et. al*.

Assumptions

The change over the study period, r, is the parameter of inferential interest: it is the fraction of the initial population remaining at the end of the study period. The null hypothesis of interest is that of no change over the study period $\mathbb{H}_0 : r = 1$, which is equivalent to a nill null hypothesis (on a log scale): $\mathbb{H}_0 : \log r = 0$. To perform Monte Carlo simulations, a data-generating mechanism wherein the parameter r intervenes, must be specified. We made the following assumptions.

1. Monitoring relies on a temporal sampling scheme having a total of T ($T \geq 3$) sampling occasions evenly spaced at times $t \in [1 : T]$;
2. each sampling occasion yields an abundance/density estimate $\hat{y_t}$ with an upper bound for the magnitude of their coefficient of variation (CV) $\mathrm{cv}_{\mathrm{y_t}}$;
3. the response variable is the ratio $\hat{p_t} = \frac{\hat{y_t}}{\hat{y_1}}$ for all $t \in [1 : T]$;
4. the observed values $\hat{p_t}$ follow a log-normal distribution; and
5. the true values are $p_t = r^{\frac{t-1}{T-1}}$.

Methods

With the above specification of the data-generating mechanism for p_t, it can be checked that (for $r > 0$):

$$\begin{cases} t = 1, \; p_1 = r^{\frac{1-1}{T-1}} = r^0 = 1 \\ t = T, \; p_T = r^{\frac{T-1}{T-1}} = r^1 = r \end{cases}$$

We, thus, assumed that data $\hat{y_t}$ are collected on each sampling occasion t: these data may be (relative) biomass, abundance, or density. The ratio of each datum to the first datum is then computed, and the dimensionless fractions $\hat{p_t}$ resulting from these simple computations will be used to infer a trend.

Taylor series expansion

Let p_t be a ratio of random variables:

$$\forall \, t, \; p_t = \frac{y_t}{y_1} \tag{1}$$

In practice $\hat{p_t} = \dfrac{\hat{y_t}}{\hat{y_1}}$ where $\hat{y_t}$ are estimates of y_t. y_1 is a reference value: it is the abundance estimate at the beginning of the study period. By definition, $p_1 = \hat{p_1} = 1$.

The variance of a ratio R of random variables n and d such that $r = \frac{n}{d}$ can be approximated by:

$$\begin{cases} \mathbb{V}[R] & = \frac{\mathbb{E}[n]^2}{\mathbb{E}[d]^2} \times \left(\frac{\mathbb{V}[n]}{\mathbb{E}[n]^2} - 2\frac{\mathrm{Cov}(n,d)}{\mathbb{E}[n]\mathbb{E}[d]} + \frac{\mathbb{V}[d]}{\mathbb{E}[d]^2} \right) \\ & = \mathbb{E}[R]^2 \times \left(\mathrm{cv}_n^2 + \mathrm{cv}_d^2 - 2\frac{\mathrm{Cov}(n,d)}{\mathbb{E}[n]\mathbb{E}[d]} \right) \\ & = \mathbb{E}[R]^2 \times (\mathrm{cv}_n^2 + \mathrm{cv}_d^2 - 2 \times \mathrm{cor}(n,d) \times \mathrm{cv}_n \times \mathrm{cv}_d) \end{cases} \tag{2}$$

where cv_n is the CV of the numerator and cv_d that of the denominator. The term $\mathrm{Cov}(n, d)$ is the covariance between the numerator and the denominator, and can be expressed as a function of a correlation $\mathrm{cor}(n, d)$. This correlation is bounded between -1 and 1. The abundance at times t depends on the abundance at the previous time step $t - 1$, and on the method to estimate abundance (e.g. distance sampling;). A positive correlation is expected because of (i) population dynamics (e.g.

abundance at time t depends on abundance at time $t-1$) and (ii) methodology (e.g. the same method was used to produce an abundance estimate at times $t-1$ and time t). The correlation between any two true abundances will result from intrinsic population dynamics: in the case of declining or increasing populations, this correlation is expected to decay the further apart in time the two abundances are. Using the same measurement method is also expected to result in a positive correlation between any two abundance estimates. We can expect (i) a positive correlation between abundance estimates, and (ii) a decay in this correlation over time. This pattern of a decaying correlation over time can be emulated with a chi-square (χ^2) random walk (Chudzicki, 2013).

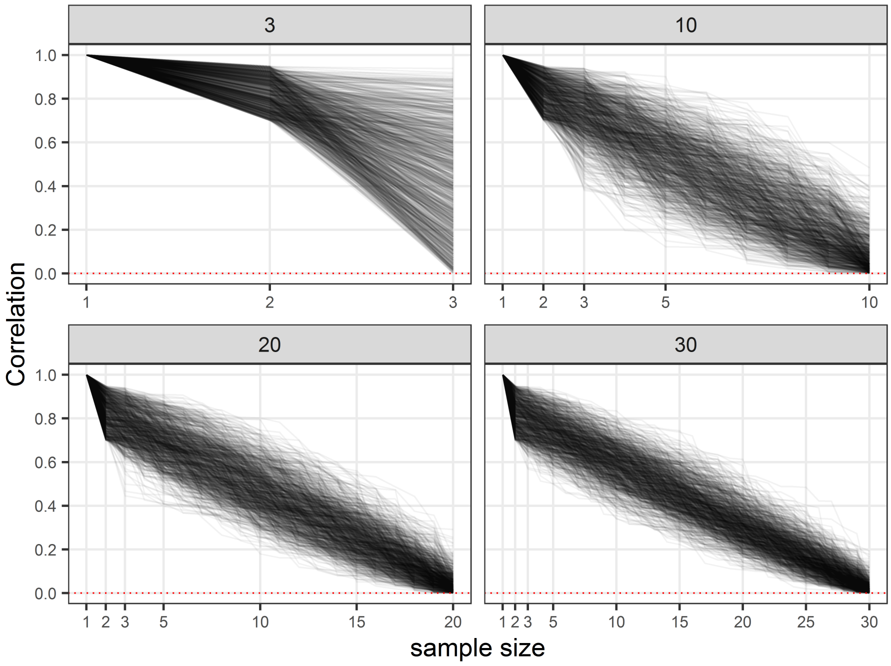

Figure 1: Modelling correlation decay between successive p_t over time. The four panels corresponds to different study length $T \in 3, 10, 20, 30$. Each line corresponds to a realization of random walk of $\chi^2(\nu = 2)$ variates. A thousand realizations are depicted on each panel. The correlation between p_1 and p_2 is arbitrarily set to lie between 0.70 and 0.95 to ensure an initial large correlation. Correlation decay is on average linear (lower panels, $T = 20, 30$) but can be very dramatic over short time-series (upper panels, $T = 3, 10$).

The CV of the ratio is:

$$\text{cv}_R = \sqrt{\frac{\mathbb{V}[R]}{\mathbb{E}^2[R]}} = \sqrt{\text{cv}_n^2 + \text{cv}_d^2 - 2 \times \text{cor}(n, d) \times \text{cv}_n \times \text{cv}_d}$$

Thus,

$$\text{cv}_{\hat{p}_t} = \begin{cases} 0 & \text{, if } t = 1 \\ \sqrt{\text{cv}_{\hat{y}_t}^2 + \text{cv}_{\hat{y}_1}^2 - 2 \times \text{cor}(\hat{y}_t, \hat{y}_1) \times \text{cv}_{\hat{y}_t} \times \text{cv}_{\hat{y}_1}} & \text{, if } t > 1 \end{cases} \quad (3)$$

The CV of all $\hat{p}_t$ depends on the CV of the reference value $\hat{y}_1$ and its correlation with the successive estimates $\hat{y}_t$.

Log-normal distribution

As in previous studies (Gerrodette, 1987; Taylor *et al.*, 2007; Jewell *et al.*, 2012), we assume the ratio of abundance/density estimates to follow a log-normal distribution:

$$p_t \sim \log\mathcal{N}(\mu_t, \sigma_t) \tag{4}$$

By definition, $\begin{cases} \mathbb{E}[p_t] &= e^{\mu_t + \frac{\sigma_t^2}{2}} \\ \mathbb{V}[p_t] &= e^{2\mu_t + \sigma_t^2} \times \left(e^{\sigma_t^2} - 1\right) = \mathbb{E}[p_t]^2 \times \left(e^{\sigma_t^2} - 1\right) \end{cases}$

Thus,

$$\begin{cases} \text{cv}_{p_t}^2 &= e^{\sigma_t^2} - 1 \Leftrightarrow \sigma_t = \sqrt{\log\left(1 + \text{cv}_{p_t}^2\right)} \\ \mathbb{E}[p_t] &= e^{\mu_t + \frac{\sigma_t^2}{2}} \Leftrightarrow \mu_t = \log \frac{p_t}{\sqrt{1 + \text{cv}_{p_t}^2}} \end{cases} \tag{5}$$

The above formulation (eq. 3-6) allows to simulate data easily: no distributional assumption on y_t nor the values of $\hat{y}_t$ are actually needed; only the range of their CVs $\text{cv}_{\hat{y}_t}$ are required in order to compute the CV of $\hat{p}_t$ (eq. 3). Then the value of r is needed to simulate a trend (eq. 6), with the sign of $\log r = \beta$ determining the direction of the trend. For example, with $T = 3$ and $r = 0.5$, the true values of p_t are 1, 0.75 and 0.5. However, the estimated $\hat{p}_t$ are observed with an error that is related to the CV of the initial abundance estimates. The coefficient of variation $\text{cv}_{\hat{p}_t}$ can be computed with eq. 3, then plugged in eq. 5 to simulate observed data.

Simulation Scenarii

We assume that the true values of p_t, the proportions of the population at time t relative to the baseline at t_1, is given by the following model:

$$p_t = r^{\frac{t-1}{T-1}} \tag{6}$$

The parameter r is represents the fraction of the initial population remaining at time $T > 1$. For example, $r = \frac{1}{2}$ means the halving of the initial population, or a 50% decrease over the study period. Taking the logarithm transform of equation 6 yields:

$$\log p_t = \log r^{\frac{t-1}{T-1}} = \frac{t-1}{T-1} \times \log r = x_t \times \beta \tag{7}$$

where $\begin{cases} x_t &= \frac{t-1}{T-1} \\ \beta &= \log r \end{cases}$. The model in eq. 7 is a linear regression with a null intercept: starting from 0 at $t = 1$, it assumes a linear trend over the study period. The parameter r is related to a trend *sensu* Link & Sauer (1997): "the percentage change [in abundance] over a specified time period".

We did not assume any relationship between true abundance and CV as in Gerrodette (1987, 1991) or Taylor *et al.* (2007). CVs may be under the control of researchers during the planning of a survey targeting a single species. However, some surveys may collect data on several species groups to augment cost-effectiveness (Lambert *et al.*, 2019): in this setting it becomes more difficult to jointly achieve a desired precision across a broad panel of species with *e.g.* different behaviour. In this setting, which is encouraged for cost-effective sampling of the marine environment, although a focal species may be a particular interest, data on other species will also be collected and the associated CVs of their estimated abundances may be viewed as random variables. Accordingly

to this view, we generated CVs for abundance estimates $\hat{y}_t$ randomly from a uniform distribution. Coefficients of variation smaller than 0.1 are not common in the literature on marine mammals (Taylor *et al.*, 2007), and we considered this lower bound to be the best precision to be realistically attainable with line transect surveys. CVs for marine mammal abundances can be large (Taylor *et al.*, 2007). To assess the impact of the precision of estimates on detecting a trend, we varied the upper bound between 0.1 and 0.5 by 0.1 increment when simulating data. Thus 5 scenarios relating to data quality (abundance/density estimates with CVs of exactly 0.1, between 0.1 and 0.2; between 0.1 and 0.3; between 0.1 and 0.4; and between 0.1 and 0.5) were investigated.

We varied the value of r, the parameter of inferential interest, between 0.5 (halving of the population over the study period T) and 0.99 (a 1% population decrease over the study period T). We did not consider declines larger than 50% as these are more readily detected (Taylor *et al.*, 2007), and we focused on ambiguous cases. Finally, the length of the study period varied between 3 and 30 by increment of 1. There were $5 \times 28 \times 38 = 5,320$ different scenarii. For each of these, $10,000$ data sets were simulated according to the following `R` code snippet:

```
make_data <- function(n, beta, upper_cv, rho = TRUE) {
  p <- seq(1, beta, length.out = n)
  cv <- runif(n, 0.1, upper_cv)
  ### correlation between measurements
  if(rho) {
    rho_start <- runif(1, 0.70, 0.95)
    eps <- rchisq(n - 1, df = 2)
    eps <- rev(cumsum(eps))/sum(eps)
    eps <- c(1, rho_start * eps)
  }
  else { eps <- rep(0, n) }
  cv_p <- sqrt(cv[1] * cv[1] + cv * cv - 2 * cv[1] * cv * eps)
  sigmalog <- sqrt(log(1 + cv_p^2))
  mulog <- log(p / sqrt(1 + cv_p^2))
  return(data.frame(t = (1:n - 1) / (n-1),
                    p_hat = c(1, rlnorm(n - 1, mulog[-1], sigmalog[-1])),
                    se = c(0.001, sigmalog[-1])
                    )
         )
}
```

Priors

The informative prior was chosen to cover *a priori* a range associated with the halving or doubling of the population over the study period: we chose a symmetric normal prior (on a logarithmic scale) centered on 0, and set the scale parameter to $\log(2)/2$. For a normal distribution, 95% of the probability mass is between $\mu - 2\sigma$ and $\mu + 2\sigma$, where μ and σ are the location and scale parameters. The weakly-informative prior was the skeptical prior of Cook *et al.* (2011). The latter uses the Cauchy distribution (a Student-t distribution with 1 degree of freedom) instead of the normal distribution (a Student-t distribution with $+\infty$ degrees of freedom) for robust inference (see also Chapter 17 in Gelman *et al.*, 2014 for a discussion of robustness) and embodies the idea that the null (no decline) is assumed *a priori* true. The location parameter of the Cauchy distribution was accordingly set to 0 and the scale parameter was set to $-\frac{\log(2)}{\tan(\pi(\xi - \frac{1}{2}))}$ where ξ is a small (skeptical) probability that β ($\log \beta$) is different from 1 (0; Cook *et al.*, 2011, page 336). This weakly-informative prior with $\xi = 0.025$ favours the hypothesis of no decline with an odds-ratio of $39 : 1$.

Null hypothesis

The null hypothesis of interest is also a nill hypothesis: $\mathbb{H}_0 : \beta = 0$. This null hypothesis corresponds to a test of no trend over the study period, or equivalently, of a stable ratio of the proportion of the initial population at the value 1. To test $\mathbb{H}_0$, both `R` functions `glm` and `bayesglm` return a two-sided p value.

```
# unregularized approach
unreg <- glm(log(p_hat) ~ -1 + x,
 family = gaussian, data = data,
 epsilon = 1e-7, maxit = 500
 )
# regularization with informative prior
strong <- arm::bayesglm(log(p_hat) ~ -1 + x,
                        family = gaussian, data = data,
prior.mean = 0,
prior.scale = log(2) / 2,
prior.df = Inf,
epsilon = 1e-7, maxit = 500
)
# regularization with weakly-informative prior
weak <- arm::bayesglm(log(p_hat) ~ -1 + x,
family = gaussian, data = data,
prior.mean = 0,
prior.scale = -log(2) / tan(pi * (0.025 - 0.5)),
prior.df = 1,
epsilon = 1e-7, maxit = 500
)
```

Difference in regularization of estimates between the Informative and Weakly-informative Priors

When we investigated type-M errors with the different regularized approach, we reported an exaggeration of effect sizes with the informative prior: estimates were too large when the true decline was also large. 'Large' in our setting means a halving of the population over the study period. The informative prior strongly penalizes estimate of $r = e^{\beta}$ that are smaller than a halving (or larger than a doubling) of the population (Figure 2). The weakly-informative prior in contrast penalizes estimates in the neighbourhood of $r \approx 1$ (that is $\beta = 0$), but not much large decline (or increase). This behaviour explains the slight underestimation of a trend with the weakly-informative prior in our Monte Carlo study.
The regularization induced with the informative prior for values of r (β) between 0.5 and 2 ($\log(\frac{1}{2})$ and $\log(2)$) is strong: this informative prior is pulling the trend estimate toward zero. Therefore, achieving statistical significance in this setting amounts to selecting the most biased estimates, that is those estimates $\hat{\beta}$ that remain far from 0. Statistical significance results in an exaggeration of effect sizes as it selects estimates that more biased compared to the unregularized approach or the regularized approach with a weakly-informative prior. This explains the higher type-M error rates of the regularized approach with an informative prior.

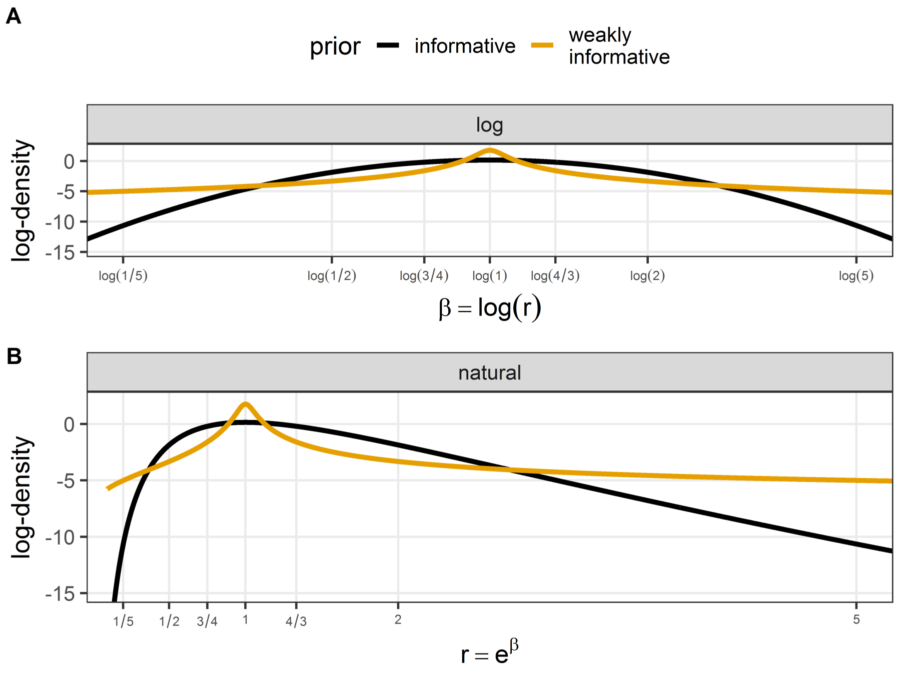

Figure 2: Log-probability density function (log-PDF) of the informative and weakly-informative priors used in regularized regression approaches. PDF are shown either on a logarithmic (left panel) or natural scale (right panel).

Case Study on the Vaquita

We illustrate our proposal using abundance data on the vaquita taken from Parsons 2018 (Table S1).

Year	t	Abundance estimate $\hat{y}_t$	$\hat{p}_t$	x_t
1997	1	567	1.000	0.000
2008	12	245	0.432	0.579
2015	19	59	0.104	0.947
2016	20	30	0.053	1.000

Table 1: Abundance estimates of the vaquita (*Phocoena sinus*) in the Sea of Cortez, Mexico.

The values $\hat{p}_t$ are derived from dividing abundance estimates $\hat{y}_t$ by the first estimate available $\hat{y}_1$. The values x_t are obtained with the formula $x_t = \frac{t-1}{T-1}$, where T is the span of the study and equals 20 years for the vaquita. The following code snippet allows to obtain three estimates of the trend: one unregularized and two regularized one.

```
# data
vaquita = data.frame(year = c(1997, 2008, 2015, 2016),
                     y = c(567, 245, 59, 30)
 )
vaquita$x <- with(vaquita, (year - min(year))/diff(range(year)))
vaquita$p_hat <- with(vaquita, y/y[1])

# unregularized approach
unreg <- glm(log(p_hat) ~ -1 + x,
 family = gaussian,
 data = vaquita,
 epsilon = 1e-7, maxit = 500
 )
# regularization with informative prior
strong <- arm::bayesglm(log(p_hat) ~ -1 + x,
                        family = gaussian,
data = vaquita,
prior.mean = 0,
prior.scale = log(2) / 2,
prior.df = Inf,
epsilon = 1e-7, maxit = 500
)
# regularization with weakly-informative prior
weak <- arm::bayesglm(log(p_hat) ~ -1 + x,
family = gaussian,
data = vaquita,
prior.mean = 0,
prior.scale = -log(2) / tan(pi * (0.025 - 0.5)),
prior.df = 1,
epsilon = 1e-7, maxit = 500
)
```

The different estimates of β can then be accessed by calling the function `print`.

```
# unregularized estimates
print(unreg, digits = 3)
# Coefficients:
#     x
# -2.49
#
# Degrees of Freedom: 4 Total (i.e. Null);  3 Residual
# Null Deviance:     14.5
# Residual Deviance: 0.574  AIC: 7.59

# regularization with informative prior
print(strong, digits = 3)
# Coefficients:
#     x
# -2.22
#
# Degrees of Freedom: 4 Total (i.e. Null);  3 Residual
# Null Deviance:     14.5
# Residual Deviance: 0.747  AIC: 8.64

# regularization with weakly informative prior
print(weak, digits = 3)
# Coefficients:
#     x
# -2.42
#
# Degrees of Freedom: 4 Total (i.e. Null);  3 Residual
# Null Deviance:     14.5
# Residual Deviance: 0.585  AIC: 7.66
```

The p value associated with the null hypothesis $\mathbb{H}_0 : \beta = 0$ can be retrieved by calling the `R` function `summary`.

```
# unregularized regression
round(summary(unreg)$coefficients, 3)
#   Estimate Std. Error t value Pr(>|t|)
# x   -2.494      0.293  -8.521    0.003

# regularization with informative prior
round(summary(strong)$coefficients, 3)
#   Estimate Std. Error t value Pr(>|t|)
# x   -2.215      0.315  -7.037    0.006

# regularization with weakly-informative prior
round(summary(weak)$coefficients, 3)
#   Estimate Std. Error t value Pr(>|t|)
# x   -2.424      0.291  -8.321    0.004
```

In the case of the vaquita data, the standard error of the estimated $\hat{\beta}$ with regularized regression with an informative prior is actually larger than that of the unregularized approach. This feature betrays a prior-data conflict: the prior tries to pull the estimate back to 0 whereas the data clearly

suggest a very large decrease of more than 50% (see also Figure 2). In contrast, the standard error of the estimated $\hat{\beta}$ with regularized regression with a weakly-informative prior is similar to, or slightly smaller than that of the unregularized approach: the prior in this case gives way to the data and the resulting regularized estimate is much closer to the unregularized one. This ability of the weakly-informative prior to let the data speak for themselves is attractive. Note that in all cases, the null hypothesis is clearly rejected both at 20% or 5% significance level.

Finally, to obtain an estimate of $\hat{r}$, and of the annual rate of change $\hat{r_a}$, we used the approach of King *et al.* (2000). Specifically, we used the estimated $\hat{\beta}$ from a regularized approach with a weakly-informative prior: we extracted the point estimate, its associated standard error and the residual degrees of freedom in order to approximate the posterior distribution of $\hat{\beta}$ with a Student-t distribution. We then generated 10^6 random values from this Student distribution, exponentiated them and took the median to estimate $\hat{r}$.

```
# regularization with weakly-informative prior
beta_hat <- sn::rst(1e6,
                    xi = summary(weak)$coefficients[1],
omega = summary(weak)$coefficients[2],
nu = nrow(vaquita) - 1
)
### overall change over the study period
r_hat <- round(exp(beta_hat), 3)
round(median(r_hat), 3)
# 0.089
### 80% confidence interval
round(quantile(r_hat, probs = c(0.1, 0.9)), 3)
#   10%   90%
# 0.055 0.143
### 80% Highest Probability Density interval
round(coda::HPDinterval(coda::as.mcmc(r_hat), prob = 0.80), 3)
# lower upper
# 0.045 0.127

### annual change averaged over the study period
r_a <- (r_hat)^(1/diff(range(vaquita$year)))
round(median(r_a), 3)
# 0.880
### 80% confidence interval
round(quantile(r_a, probs = c(0.1, 0.9)), 3)
#   10%   90%
# 0.858 0.903
### 80% HPD interval
round(coda::HPDinterval(coda::as.mcmc(r_a), prob = 0.80), 3)
# lower upper
# 0.858 0.901
```

References

Chudzicki, D. (2013) A Bayesian Model for a Function Increasing by Chi-Squared Jumps (in `Stan`). blog.

Cook, J., Fúquene, J. & Pericchi, L. (2011) Skeptical and Optimistic Robust Priors for Clinical Trials. *Revista Colombiana de Estadistica*, **34**, 333–345.

Gelman, A., Carlin, J.B., Stern, H.S., Dunson, D.B., Vehtari, A. & Rubin, D.B. (2014) *Bayesian Data Analysis*. CRC Press, 3rd edition.

Gerrodette, T. (1987) A Power Analysis for Detecting Trends. *Ecology*, **68**, 1364–1372.

Gerrodette, T. (1991) Model for Power of Detecting Trends - A Reply to Link and Hatfield. *Ecology*, **72**, 1889–1892.

Jewell, R., Thomas, L., Harris, C., Kaschner, K., Wiff, R., Hammond, P. & Quick, N. (2012) Global Analysis of Cetacean Line-Transect Surveys: Detecting Trends in Cetacean Density. *Marine Ecology Progress Series*, **453**, 227–240.

King, G., Tomz, M. & Wittenberg, J. (2000) Making the Most of Statistical Analyses: Improving Interpretation and Presentation. *American Journal of Political Science*, **44**, 341–355.

Lambert, C., Authier, M., Dorémus, G., Gilles, A., Hammond, P., Laran, S., Ricart, A., Ridoux, V., Scheidat, M., Spitz, J. & Van Canneyt, O. (2019) The Effect of a Multi-Target Protocol on Cetacean Detection and Abundance Estimation in Aerial Surveys. *Royal Society Open Science*, **6**, 1–17.

Link, W. & Sauer, J. (1997) New Approaches to the Analysis of Population Trends in Land Birds: Comment. *Ecology*, **78**, 2632–2634.

Parsons, E.C.M. (2018) *"Dark times lie ahead of us and there will be a time when we must choose between what is easy and what is right"* - The Sad Case of Vaquita, the Trump Administration and the Removal of Protections for Whales and Dolphins. *Journal of Environmental Studies and Sciences*.

Taylor, B., Martinez, M., Gerrodette, T., Barlow, J. & Hrovat, Y. (2007) Lessons from Monitoring Trends in Abundance of Marine Mammals. *Marine Mammal Science*, **23**, 157–175.
